# Effect of intranasal administration of *Erigeron annuus* and *Carthamus tinctorius* extracts in a rat model of olfactory dysfunction induced by 3-methylindole

**DOI:** 10.1371/journal.pone.0325429

**Published:** 2025-06-10

**Authors:** Myeongguk Jeong, Hyeokjin Kwon, Yeeun Kim, Kyung-Yae Hyun, Yeongdon Ju, Go-Eun Choi, Chulhun L. Chang

**Affiliations:** 1 Department of Biomedical Laboratory Science, College of Health Sciences, Catholic University of Pusan, Busan, Republic of Korea; 2 Department of Clinical Laboratory Science, Dong-Eui University, Busan, Republic of Korea; 3 Sound -Phonics Institute, Dong-Eui University, Busan, Republic of Korea; 4 Department of Biomedical Laboratory Science, Gimcheon University, Gimcheon, Republic of Korea; 5 Department of Laboratory Medicine, School of Medicine, Pusan National University, Yangsan, Republic of Korea; 6 Department of Laboratory Medicine, Pusan National University Yangsan Hospital, Yangsan, Republic of Korea; Shiraz University of Medical Sciences, IRAN, ISLAMIC REPUBLIC OF

## Abstract

There are many factors that can cause olfactory dysfunction, including upper respiratory tract viral infections, non-inflammatory respiratory diseases, trauma, and current treatments such as medications and surgery can have adverse effects and may not respond. Therefore, we aimed to develop a natural product-based adjunctive treatment strategy for olfactory dysfunction that is safe and has minimal adverse effects. We investigated the effects of extracts from *Erigeron annuus* and *Carthamus tinctorius*, which have demonstrated anti-apoptotic, neuroprotective, and anti-inflammatory activities, on 3-methylindole-induced olfactory dysfunction. A 3-methylindole-induced olfactory dysfunction model rat was established and olfactory dysfunction was treated with intranasal administration of *Erigeron annus* extract (EAE) and *Carthamus tinctorius* extract (CTE) or their combination. After 3 weeks, alterations in food-finding tests and OMP expression in olfactory bulb and olfactory epithelium were assessed. Comparing the food finding test, the EAE + CTE group had a significant decrease in food finding time compared to the vehicle group. IHC and Western blot analyses showed that OMP expression in the olfactory bulb was significantly increased in the EAE + CTE group compared to the vehicle group. Western blot analysis of olfactory epithelial tissue also showed a significant increase in OMP expression. Intranasal administration of EAE + CTE alleviated 3-methylindole-induced olfactory dysfunction.

## Introduction

The olfactory system, crucial for animal survival, is among the oldest sensory systems. Olfactory dysfunction can impact health and quality of life by diminishing food enjoyment, lowering appetite, and limiting sensory input from the surroundings [1–3]. A recent study discovered that approximately 20% of people experience olfactory dysfunction, with 15% having hyposmia and 5% having anosmia [4,5]. There are many factors that contribute to olfactory dysfunction, including upper respiratory viral infections, nasal inflammation, non-inflammatory respiratory diseases, and trauma [6,7]. Despite known treatments for olfactory dysfunction, such as vitamin A steroid therapy, some cases do not respond to either medication or surgery [8–10]. Therefore, there is a need to develop alternative treatments for olfactory dysfunction.

*Erigeron annuus* (EA), a member of the Asteraceae family, has traditionally been used to treat ailments such as enteritis and acute gastroenteritis due to its flavonoids and coumarins [11–13]. Previous research has shown that extracts from EA possess anti-apoptotic and neuroprotective properties [14,15]. *Carthamus tinctorius* (CT) belongs to the Asteraceae family and has traditionally been used to treat cardiovascular disease, connective tissue disorders, blood circulation issues, and skeletal disorders [16–18]. In addition, CT has demonstrated antithrombotic, analgesic, antitumor, anti-inflammatory, and neuroprotective effects in previously reported studies [19–21].

The 19 kDa olfactory marker protein (OMP) is a key protein expressed in the olfactory system. [22]. OMP serves various functions, including its presence in cilia, dendrites, somas, and axons, as well as its interactions with other proteins involved in odor signaling [23]. In both olfactory receptor neurons and glomeruli within the olfactory bulb, OMP contributes to the refinement of the glomerular map and axon pruning [23,24]. It is essential to develop a neural network that selectively encodes odor information [25–27].

This study evaluates the olfactory function-improving effects of EA and CT extracts through OMP expression and behavioral analysis in a rat model of 3-methylindole-induced olfactory dysfunction and aims to provide a basis for the development of natural product-based olfactory dysfunction treatment strategies.

## Materials and methods

### *E. annuus* extract (EAE) and *C. tinctorius* extract (CTE)

The extract was prepared with a minor modification to a previously described method [28]. *E. annuus* and *C. tinctorius* were purchased from Daoom International (Hanam, Republic of Korea) and dried in a 40 °C dry oven. The dried plants were ground and soaked in distilled water at a 1:20 ratio, then incubated overnight at 60 °C. The extract was then filtered through a 0.22 μm vacuum filter, concentrated with a rotary evaporator, and lyophilized to obtain a powdered extract. The powdered extract was stored at 4 °C, away from light, until further used.

### Animals

Male Sprague-Dawley (SD) rats, six weeks old, were acquired from Samtako (Osan, Republic of korea). The rats were kept in plastic cages with corn bedding, maintained at 22 ± 2°C, 50 ± 5% humidity, and a 12 h light/dark cycle. Food and water were available ad libitum, and the rats underwent a one-week acclimatization phase prior to the experiment. The Animal Experimentation Ethics Committee of Dong-Eui University approved this study and was conducted according to the ethical guidelines of the committee (approval number: R2022-030). We tried to minimize suffering for all animals. On the last day of the experiment, the sacrifices were euthanized with CO_2_ without anesthesia.

### 3-methylindole injection and extract intranasal administration

SD rats were randomly assigned to 6 groups: Normal group, Vehicle group, EAE 5%, CTE 5% group, EAE + CTE 5% group, 0.25 mg/kg Dexamethasone (DEX) group. To induce olfactory dysfunction, the experimental groups, except for the normal group, received intraperitoneal injections of 3-methylindole dissolved in corn oil at a dose of 300 mg/kg. The normal group was administered intraperitoneally only corn oil without 3-methylindole in the same amount as the vehicle group. After establishing the olfactory dysfunction model, 5% EAE, 5% CTE, 5% EAE + CTE, and 0.25 mg/kg DEX were all dissolved in PBS and prepared fresh before treatment. For intranasal administration, rats were lightly anesthetized with isoflurane. Rats were placed in a supine position close to 180° and administered nasally at a volume of 40 μl per nostril and held in this position for 10 sec. The Vehicle group received 40 µl of PBS only. Intranasal administration was treated daily for 3 weeks.

### Behavioral test

The food-finding test was used to determine if olfactory function was recovered. After all treatments were completed, the rats were not fed for 24 h and were placed in a new cage with bedding and 2 g of food pellets buried under the bedding. Rats were observed for up to 5 min; If the rat did not find the food pellet within this time, the test was terminated. The bedding and food pellets were replaced after each test.

### Immunohistochemistry

After completing all experiments, the rats were euthanized and dissected for immunohistochemical staining. The olfactory bulbs were collected, preserved in 10% paraformaldehyde, and paraffin blocks were prepared. Paraffin blocks were cut to 4 μm thickness for slide sections and de-paraffinized and rehydrated using xylene and various concentrations of alcohol. Immunohistochemistry staining was performed with the VECTASTAIN ABC kit (PK-6101, Vector Laboratories, Newark, CA, USA). Blocking Solution (Vector Laboratories, Newark, CA, USA) was used to block the activity of the endogenous enzyme. The sections were incubated overnight at 4°C with a monoclonal antibody against Anti-Olfactory Marker Protein (1:800, abcam, Cambridge, UK). Following PBS washing, after incubation with biotinylated secondary antibody, the sections were exposed to peroxidase substrate solution (SK-4100, Vector Laboratories, Newark, CA, USA) until staining intensity was reached. Counterstaining was performed using hematoxylin. Measurement points for all sections were taken from three different sections. Images of the sections were taken with a Leica DMi1 (Leica, Wetzlar, Germany) and the DAB-stained regions were measured using Image J software (v1.53, Bethesda, MD, USA)

### Western blotting

To extract total protein, the sample was homogenized with RIPA buffer, which included protease inhibitors. The supernatant was then centrifuged at 17 000 × g for 15 min at 4°C. Proteins were quantified using the Bicinchoninic Acid (BCA) Protein Assay Kit and loaded at the same concentration. Proteins were then separated by electrophoresis on 12% polyacrylamide gels. Primary antibodies included rabbit anti-GAPDH (1:10,000, Cell Signaling Technology) and rabbit anti-OMP (1:1,000, Abcam), which were applied overnight at 4°C. Following a wash using PBST, the membrane was incubated for 1 h with a goat anti-rabbit secondary antibody that was conjugated to horseradish peroxidase (1:1,000, Cell Signaling Technology). They were then treated with an enhanced chemiluminescence kit and finally visualized. (ChemiDoc XRS+ system, Bio-Rad, Hercules, CA, USA).

### Statistical analysis

The data are presented as mean ± standard deviation (SD). Statistical analyses were conducted with GraphPad Prism 6 software (v6.01, San Diego, CA, USA). Data were analyzed by one-way ANOVA followed by Dunnett’s multiple comparisons test. All results were obtained from at least three independent repetitions and are expressed as the mean ± SD(n = 3) from a single experiment. A *P* value of less than 0.05 was deemed statistically significant.

## Results

### Food finding test

**Fig 1:**
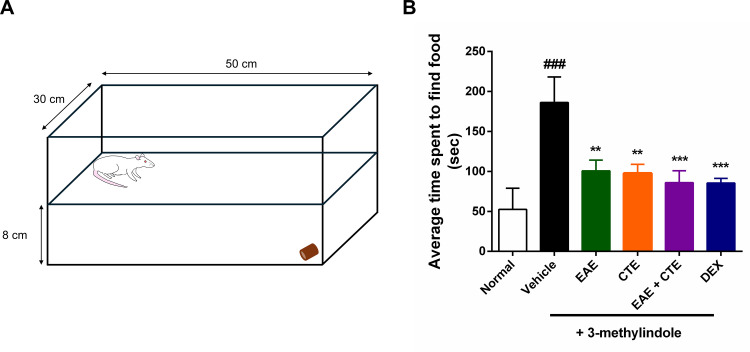
Olfactory function test graphical summary and results. (A) Graphical overview of the food-finding test. (B) All groups, except the normal group, receive 3-methylindole intraperitoneally to establish a model of olfactory dysfunction, followed by intranasal treatment with 5% EAE, 5% CTE, 5% EAE + CTE, and dexamethasone. The food-finding test is conducted on the last day after all treatments. The bar graph shows the mean ± SD. ^###^*p* < 0.001 Compared to the normal group. ^***^p < 0.001 Compared to the vehicle group.

The effect of EAE and CTE extracts or their combination was evaluated by food finding test on the last day of all treatments. Rats were given 24 h without food and timed by burying a food pellet under the end bedding opposite the starting point (Fig 1A). The Normal group found food in an average of 52.5 ± 22.8 s. The vehicle group treated with 3-methylindole significantly increased the time to find food, averaging 186.2 ± 27.6 s. Food finding time after extract or dexamethasone treatment was significantly reduced to 100.7 ± 11.6 s, 97.75 ± 9.6 s, 86 ± 12.8 s, and 85.5 ± 5.0 s for the EAE group, CTE group, EAE + CTE group, and DEX group (Fig 1B).

### Immunohistochemistry

**Fig 2:**
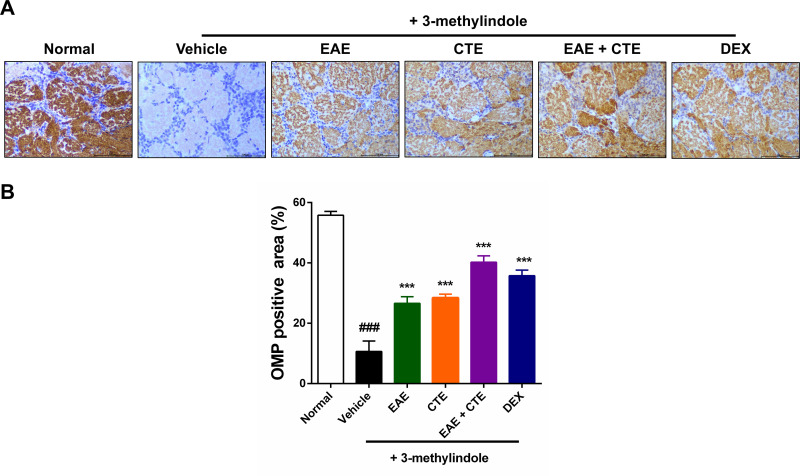
Immunohistochemical staining results for OMP in olfactory bulb. (A) Representative immunohistochemistry image of olfactory bulb tissue from rat stained with OMP (×400). (B) Quantification of OMP immunohistochemistry stain-positive areas in rat olfactory bulb. The bar graph shows the mean ± SD. ^###^*p* < 0.001 Compared to the normal group. ^***^p < 0.001 Compared to the vehicle group.

To investigate whether EAE, CTE, and their combination restores 3-methylindole-induced olfactory dysfunction, immunohistochemical staining of OMP was performed on olfactory bulb tissue (Fig 2A). Compared with the vehicle group, 3-methylindole treatment significantly decreased OMP expression in the olfactory bulb of the normal group. Intranasal administration of 5% EAE, 5% CTE, 5% EAE + CTE, and DEX significantly increased the OMP-positive area in the olfactory bulb (Fig 2B).

### Western blotting

**Fig 3:**
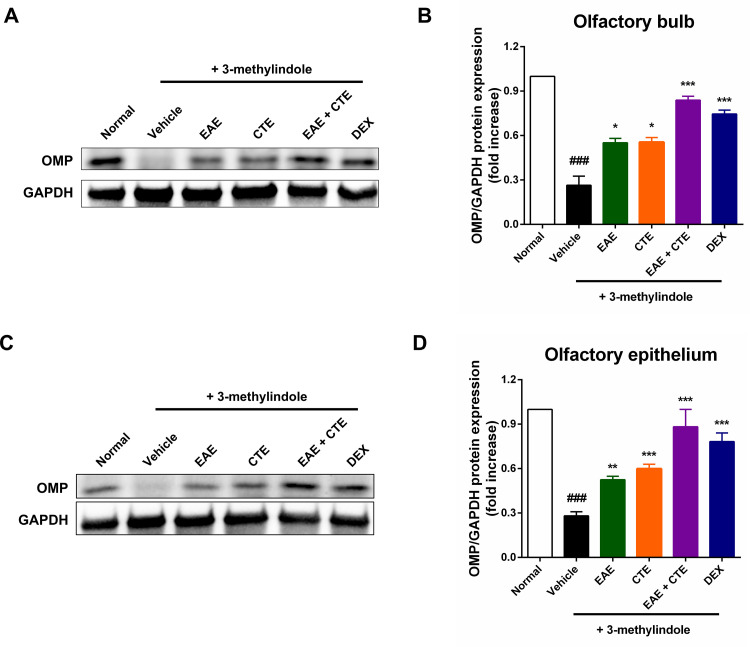
Western blotting for OMP expression using olfactory bulb and olfactory epithelium. (A) OMP expression in the olfactory bulbs of rats treated with different extracts, assessed by western blotting analysis. (B) Quantification of olfactory bulb OMP expression. (C) OMP expression in olfactory epithelium of mice treated with different extracts assessed by Western blotting analysis. (D) Quantification of olfactory epithelial OMP expression. The bar graph shows the mean ± SD. ^###^*p* < 0.001 Compared to the normal group. ^*^p < 0.05; **p < 0.01; ^***^p < 0.001 Compared to the vehicle group.

To investigate whether EAE, CTE extract, or their combination alleviates 3-methylindole-induced olfactory dysfunction, we examined the expression of OMP in olfactory bulb tissue and whole olfactory epithelium by western blotting (Fig 3A, C). The vehicle group treated with 3-methylindole alone had a significant decrease in OMP expression in olfactory bulb and olfactory epithelium. However, OMP expression of EAE, CTE, EAE + CTE, and DEX was significantly increased in the olfactory bulb and olfactory epithelium after intranasal administration (Fig 3B, D).

## Discussion

Although there are several treatments for olfactory dysfunction, they are limited by a variety of etiologies and side effects [29,30]. Various interventions are available; however, glucocorticoids are commonly prescribed. Glucocorticoid receptors in the olfactory neuroepithelium have been identified on the cilia of olfactory receptor neurons, glandular cells of Bowman’s glands, and axonal bundles [31]. However, the mechanisms underlying olfactory dysfunction remain unclear. Topical steroids have shown some efficacy in treating olfactory deficits associated with nasal disease [9,32], most likely because of their anti-inflammatory properties and ability to reduce nasal obstruction. However, it has limited effectiveness in addressing sensory nerve deficits or promoting olfactory regeneration.

3-methylindole was administered intraperitoneally to establish a rat model of olfactory dysfunction. 3-methylindole is a tryptophan metabolite that causes pulmonary, hepatic, and olfactory toxicity [33]. Harmful metabolic byproducts, including 3-methyleneindolenine generated by cytochrome P450, are known to cause toxicity to the olfactory epithelium [34,35]. 3-methylindole is believed to interfere with signal transduction to bulbous areas involved in olfactory responses. In other terms, it interferes with the axonal transport pathway from the olfactory neuroepithelium to the olfactory bulb [36]. It has been reported that injection of 300 or 400 μg/g of 3-methylindole decreases the number of olfactory sensory neurons in the olfactory neuroepithelium by about 80% [37]. A study by Li et al. found that the histological thickness of the olfactory epithelium decreased after administration of 3-methylindole. After 28 days, the thickness had only recovered to about half of its original value. In addition, persistent impairment of olfactory behavior was reported that lasted for 4 weeks [38]. This study also showed that the olfactory dysfunction induced by 3-methylindole did not return to normal after 21 days.

To determine whether 3-methylindole causes olfactory dysfunction, a food-finding test was performed. Our study found that the vehicle group receiving 300 mg/kg 3-methylindole showed a significant increase in food-finding time; however, nasal administration of EAE + CTE decreased this time. This research demonstrated that the food-finding test is a practical and efficient method for assessing overall olfactory function in a rat model. This simple, rapid test requires minimal training, no costly equipment, and can be easily conducted in most laboratory settings [39].

We assessed OMP expression levels in olfactory bulbs and olfactory epithelium along with food-finding time measurements. OMP plays an important role in olfactory receptor neuron development, and is an important marker of olfactory receptor neuron maturation [24,26]. Olfactory receptor neurons extend in the nasal mucosa, and their axons extend to the olfactory bulb. Within the olfactory bulb, olfactory receptor neurons establish synapses with mitral and tufted cells, both of which serve as second order neurons in the olfactory system. From this point, the axon travels along the lateral olfactory tract, reaching different areas of the brain, including the olfactory tubercle, pre-olfactory nucleus, piriform cortex, and hypothalamus. This elaborate process highlights the sophistication and accuracy of the olfactory neural pathway [40]. Our results showed that intraperitoneal infection 3-methylindole completely decreased OMP expression in olfactory bulb and olfactory epithelium. The research conducted by Kim et al. showed that 300 μg/g of 3-methylindole completely decreased OMP expression in olfactory bulb and olfactory epithelium, which is consistent with our findings [37]. However, OMP expression in the olfactory bulb and olfactory epithelium of the EAE + CTE group was significantly increased after intranasal administration.

Although this study showed that EAE and CTE were effective in improving olfactory function, there are features that may explain these beneficial effects. *E.annuus* and *C.tinctorius* contain compounds that have been shown to have antioxidant and neuroprotective effects. Caffeic acid contained in *E.annuus* has been shown to protect nerve cells from oxidative stress and exhibit neuroprotective effects [15]. Similarly, *C.tinctorius* has been demonstrated to have free radical scavenging activity and neuroprotective effects [41,42]. Considering that 3-methylindole induces toxicity partly through oxidative damage and free radical generation [33], the antioxidant activity of *E.annuus* and *C.tinctorius* may counteract this neurotoxicity. Furthermore, increased expression of OMPs indicates recovery of the olfactory sensory neuron population. The observed increase in OMP expression in both the olfactory bulb and the epithelium supports the idea that EAE and CTE may promote neuronal survival, differentiation, or regeneration through neuroprotective mechanisms. This is based on the function of OMP, a marker of mature olfactory sensory neurons, and the neuroprotective mechanism demonstrated in EAE and CTE.

This study has a few limitations. First, the morphology of the olfactory epithelium has not been determined; therefore, the morphological parameters of the olfactory epithelium need to be evaluated. We also need to investigate by what mechanisms the extracts used alleviate olfactory dysfunction. Second, *E.annuus* and *C.tinctorius* extracts were obtained using various methods, and differences in the composition of may arise when different solvents, such as methanol and water, are used. Comparing the efficacy of extracts obtained using different extraction methods may further clarify their effects. Third, toxicological studies on the intranasal administration of EAE and CTE are lacking. Although no adverse effects were observed in this study, further research is required to confirm their safety.

Our results showed that intranasal administration of EAE and CTE in a rat model of olfactory dysfunction induced by 3-methylindole could significantly increase OMP expression in the olfactory bulb and olfactory epithelium, thereby alleviating olfactory dysfunction.

## Conclusions

Intranasal co-administration of EAE and CTE restored olfactory function in a rat model of 3-methylindole-induced olfactory dysfunction, as evidenced by decreased food finding time and increased expression of OMP in the olfactory bulb and epithelial cells. The antioxidant properties of this co-administration appear to attenuate oxidative neuronal damage, and the neuroprotective components promote survival and regeneration of OMP positive neurons, suggesting therapeutic potential for olfactory dysfunction.

## Supporting information

S1 raw images Western blotting whole membrane images https://doi.org/10.6084/m9.figshare.28613384.v1.
(PDF)
