# Supplementary material for: Effect of intranasal administration of Erigeron annuus and Carthamus tinctorius extracts in a rat model of olfactory dysfunction induced by 3-methylindole
Source: PLoS One. 2025 Jun 10;20(6):e0325429. doi: 10.1371/journal.pone.0325429 (PMC12151353; doi:10.1371/journal.pone.0325429)
Supplement: S1 raw images — Western blotting whole membrane images https://doi.org/10.6084/m9.figshare.28613384.v1. (PDF) [file pone.0325429.s001.pdf]

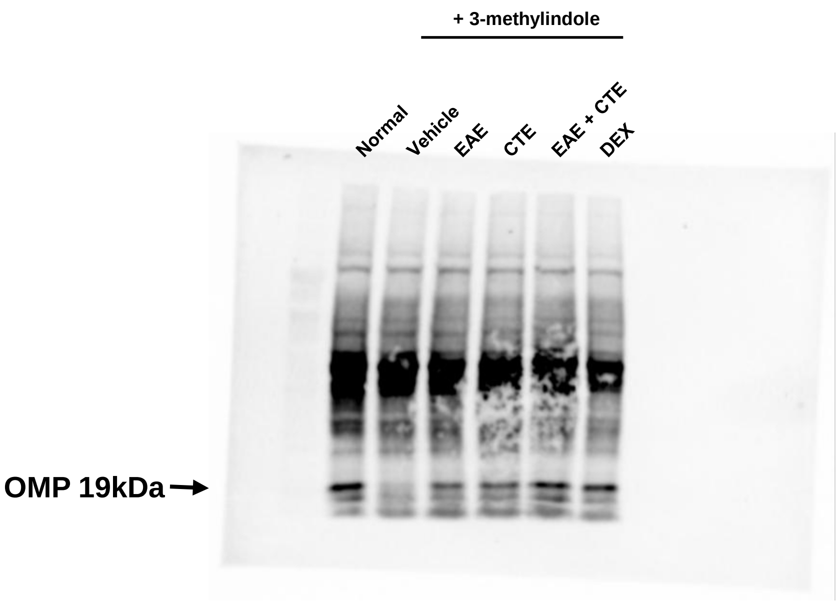

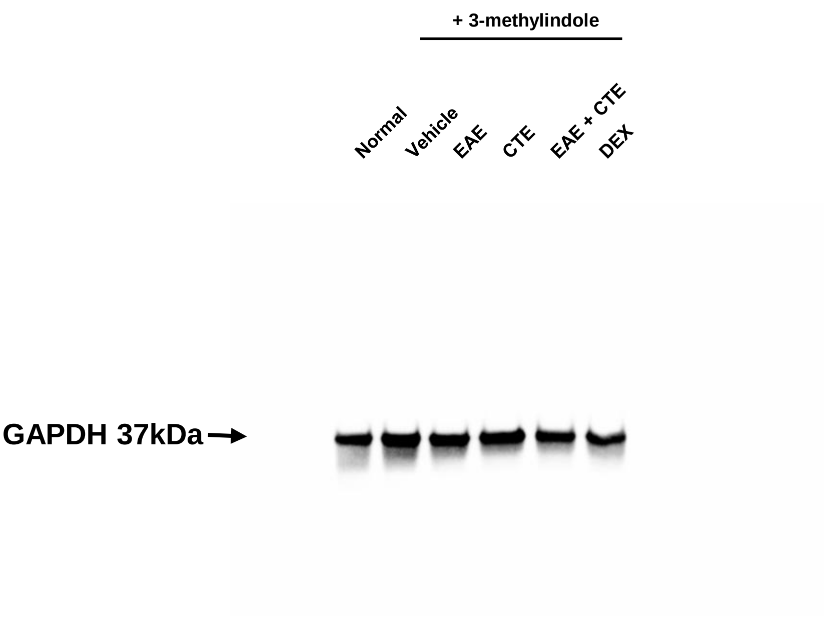

Fig s1. Original image of western blot for olfactory marker protein (OMP) expression in mouse olfactory bulb.

Fig s2. Original image of western blot for GAPDH expression in mouse olfactory bulb.

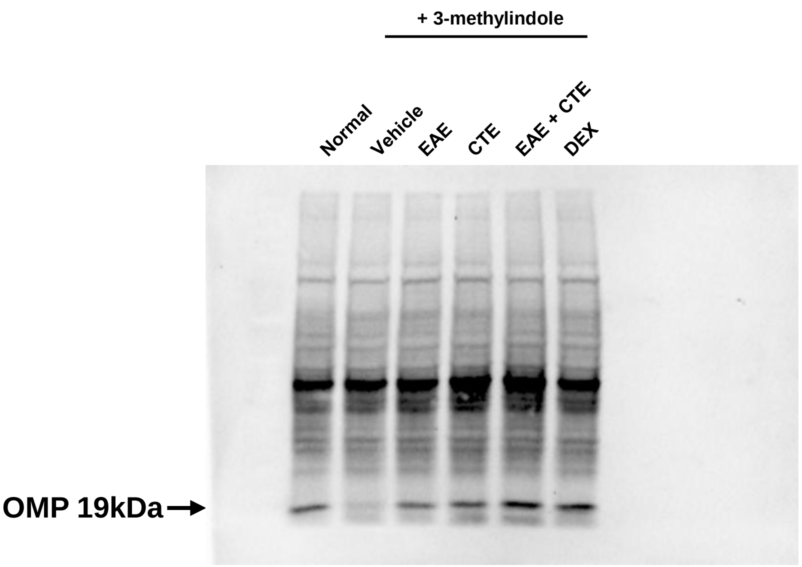

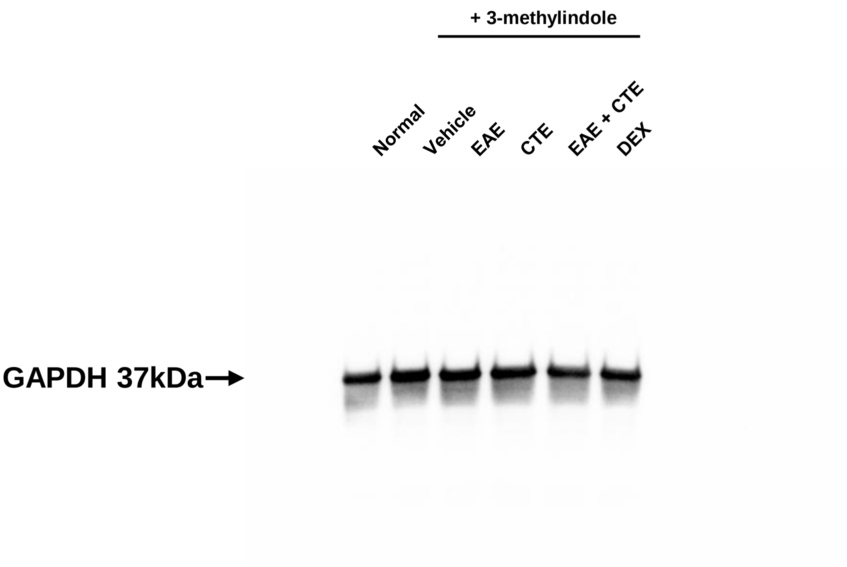

Fig s3. Original image of western blot for olfactory marker protein (OMP) expression in mouse olfactory epithelium.

Fig s4. Original image of western blot for GAPDH expression in mouse olfactory epithelum.
